# Expression of Concern: Caveolin‐1 Is Required for Contractile Phenotype Expression by Airway Smooth Muscle Cells

**DOI:** 10.1111/jcmm.71205

**Published:** 2026-05-18

**Expression of Concern:** R. Gosens, G. L. Stelmack, S. T. Bos, G. Dueck, M. M. Mutawe, D. Schaafsma, H. Unruh, W. T. Gerthoffer, J. Zaagsma, H. Meurs, and A. J. Halayko, “Caveolin‐1 Is Required for Contractile Phenotype Expression by Airway Smooth Muscle Cells,” *Journal of Cellular and Molecular Medicine* 15, no. 11 (2011): 2430–2442, https://doi.org/10.1111/j.1582‐4934.2010.01246.x.

This Expression of Concern is for the above article, published online on 30 December 2010 in Wiley Online Library (wileyonlinelibrary.com), and has been issued by agreement between the journal Editor‐in‐Chief, Stefan N. Constantinescu; the Foundation for Cellular and Molecular Medicine; and John Wiley and Sons Ltd. The Expression of Concern has been agreed following concerns raised by a third‐party regarding image duplication. An investigation identified that the sm‐MHC bands and Beta actin bands presented in Figure 1A of this article are duplicated from a figure in another article published earlier by some of the same authors. Further analysis revealed evidence of splicing between the two Caveolin‐1 bands and the two sm‐MHC bands shown in Figure 1A. The authors cooperated with the investigation and provided some supporting data. However, while the material supplied was consistent with the reported results and conclusions, it did not correspond to the images as published. As a result, the journal has issued this Expression of Concern to alert readers to these unresolved issues.

